# Portal and mesenteric venous calcification in patient with advanced cirrhosis

**DOI:** 10.1097/MD.0000000000030766

**Published:** 2022-10-07

**Authors:** Bing Pan, ShaoCheng Lyu, Qiang He

**Affiliations:** a Department of Hepatobiliary Surgery, Beijing Chaoyang Hospital, Capital Medical University, Beijing, China.

**Keywords:** calcification, case report, liver cirrhosis, liver transplantation, portal hypertension, portal vein

## Abstract

The incidence of portal and mesenteric venous calcifications in patients with cirrhosis has rarely been reported. It is also very difficult to determine the vascular lesions in preoperative imaging examination. The liver cirrhosis patients associated with portal venous calcification have high postoperative complications and mortality, but poor prognosis.

**Patient concerns:**

We present a patient (45-year-old male), who was admitted to the hospital with liver cirrhosis and portal hypertension associated symptoms.

**Diagnoses:**

Abdominal imaging computed tomography confirmed the presence of calcification in the portal vein system.

**Interventions:**

The patient underwent allogeneic liver transplantation.

**Outcomes:**

The patient died 2 days after liver transplantation.

**Lessons:**

Calcification in the portal vein system is extremely rare, and always occurs in patients with long-standing liver cirrhosis with portal hypertension gastroesophageal varices and splenomegaly. The presence of portal vein calcification on computed tomography may be a sign of portal vein thrombosis, which may result in a difficult transplantation, and poor prognosis.

## 1. Introduction

The incidence of portal and mesenteric venous calcifications in patients with cirrhosis has rarely been reported. It is also very difficult to determine the vascular lesions in preoperative imaging examination. The liver cirrhosis patients associated with portal venous calcification have high postoperative complications and mortality, but poor prognosis. Here, we reported two recent cases with evidence of calcifications in the portal venous system confirmed by computer tomography.

## 2. Patient concerns

A 45-years-old man was admitted for fever of 3 days duration and was diagnosed with acute cholangitis, biliary cirrhosis. He has congenital cystic dilatation of the intrahepatic bile duct but has not been examined and treated. 30 years ago, he underwent splenectomy and venous devascularization due to upper gastrointestinal bleeding caused by portal hypertension-related liver cirrhosis. In the past 20 years, he had repeated gastrointestinal bleedings and underwent endoscopic varicose ligation combined with embolization for hemostasis. In the past 1 year, the patient has repeatedly had fever with chills, abdominal pain, diarrhea, jaundice, and other symptoms. He denied smoking and alcohol abuse.

## 3. Diagnosis

**Figure 1.**
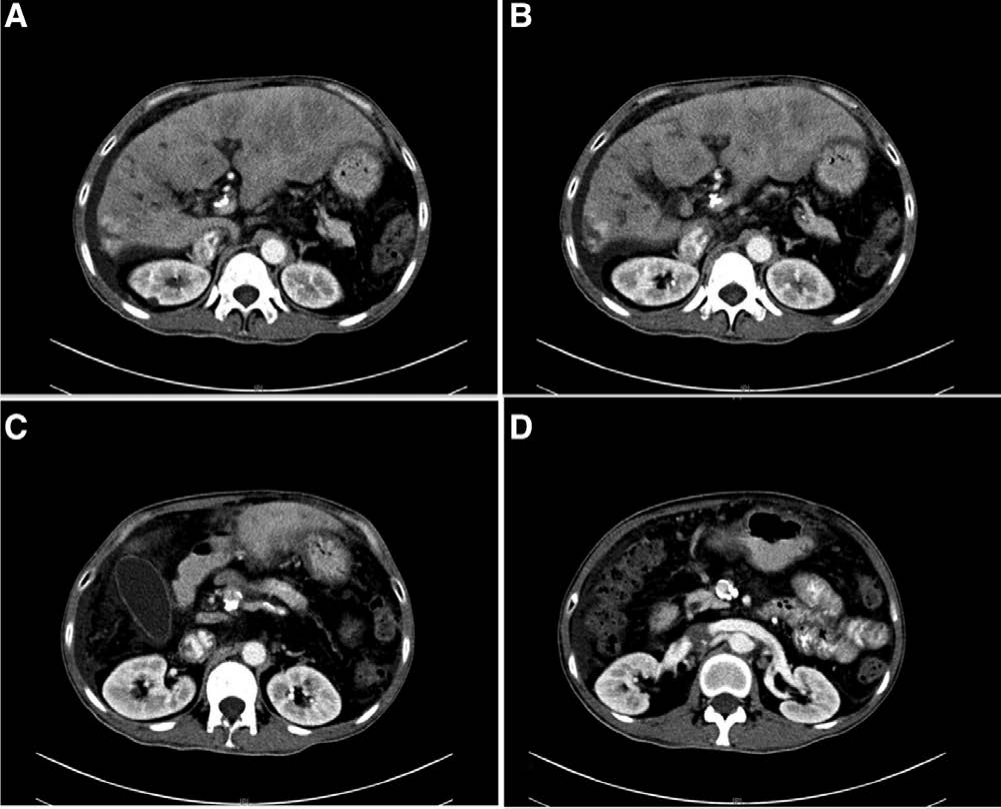
The contrast-enhanced CT of the abdomen of patient. (A and B) Enhanced CT of the abdomen suggests multiple dilation of the intrahepatic bile ducts because of Caroli disease, and liver cirrhosis and portal hypertension. (C and D) Multiple calcifications in the running area of the portal vein and superior mesenteric vein. CT = computed tomography.

Physical examination was essentially negative except for body temperature as high as 38.7°C. Lab-examination showed: white blood cell = 15.7 × 10^9^/L, neutrophil (NE)% = 77.8%, hemoglobin = 122 g/L, platelet = 207 × 10^6^/L; albumin = 27.4 g/L, alanine aminotransferase = 112 U/L, aspartate aminotransferase = 80 U/L, total bilirubin 30.3 µmol/L, direct bilirubin = 10.2 µmol/L; blood ammonia = 106 µmol/L; prothrombin time = 17.4 seconds, prothrombin time activity = 76.2%, international normalized ratio = 1.16, activated partial thromboplastin time = 30.0 seconds; carbohydrate antigen 19-9 150.20 U/L, carbohydrate antigen 12-5 174.50 U/L, hepatitis related tests are negative. Abdominal enhanced computed tomography (CT) indicated multiple dilation of intrahepatic bile duct, which is consistent with the manifestations of Caroli disease (Fig. 1A and B), and calcifications in the portal and superior mesenteric were detected (Fig. 1C and D), liver cirrhosis with portal hypertension. His liver function was Child-Pugh A grade and MELD 6 points.

## 4. Interventions

Based on comprehensive considerations, the patient met the indications for liver transplantation, and was reviewed and approved by the hospital ethics committee to undergo the allogeneic modified piggyback liver transplantation on January 23, 2021. The surgical procedure was complicated and took 25 hours, including 7 hours in the anhepatic phase. The cold ischemia-time of the donor liver was 16.5 hours. After a difficult hepatectomy, the portal vein was found to be calcified and thrombosed. Attempted at direct intraluminal dissection and thrombectomy failed to reestablished patency. Finally, the portal vein stent was placed, and portal flow was reestablished. Intraoperative blood loss is estimated to be 16,000 mL, 6500 mL autologous blood transfusion, 3600 mL suspended red blood cells, and 2400 mL fresh frozen plasma.

## 5. Outcomes

Postoperative ultrasound of the transplanted liver vessels showed that the inner diameter of the portal vein was 1.0 cm and the flow rate was 18.0 cm/s. With long operation time, excessive bleeding, long cold ischemia time of the donor liver, and poor portal vein condition, the patient died of transplanted liver failure, kidney failure, and heart failure 2 days after surgery.

## 6. Conclusion

Calcification in the portal vein system is extremely rare, and always occurs in patients with long-standing liver cirrhosis with portal hypertension gastroesophageal varices and splenomegaly.^[1]^ For revealing portal vein and its tributaries, abdominal enhanced CT could improve the positive rate, and is the most sensitive examination, and showed the location and direction of portal venous calcification.^[2,3]^ The distinctive radiographic feature of portal venous calcification is the presence of radiodensity which correspond to the course of the vein.^[4]^ Minimal calcification may be frequently neglected on plain film radiography and pathological examination.

Calcium could be deposited either in thrombus or as in the vessel wall. The mechanical stress may result in sclerosis and calcification with the thickened and media of the vein. Since 1943, Moberg^[5]^ reported the first case of portal vein calcification, <50 documented cases have been described in the English-language literature. The calcified lesions occurred in the portal vein in 100% of patients, the splenic vein in 62%, the superior mesenteric vein in 33%, and the inferior mesenteric vein in 0.^[6]^ Repeated thrombus formation and recanalization may be the main etiologic factor in the formation of calcification. The predisposing factors for the deposition of calcified thrombus in the portal vein well, included visceral infections affected by pancreatitis and cholangitis, history of abdominal surgery, malignant diseases, and hematological abnormalities.^[7]^ It was found by Verma^[4]^ a high operative mortality associated with calcifications in portal venous system in patients during liver transplantation because of preoperatively undiagnosed thrombosis of the portal venous system. And the presence of portal vein calcification on CT may be a sign of portal vein thrombosis, which may result in a difficult transplantation. The calcification of portal venous system with associated thrombosis is a significant finding and more attention should be devoted to detecting in patients undergoing liver transplantation. Identification of patients at high risk may provide information for prospective planning, rational distribution of organs, and a safer operation.

Calcification in the portal vein system is extremely rare, and always occurs in patients with long-standing liver cirrhosis, portal hypertension. Abdominal enhanced CT showed the location and direction of portal venous calcification is the most sensitive examination. Calcium could be deposited either in thrombus or vessel wall. The mechanical stress may result in sclerosis and calcification with the thickened and media of the vein. Repeated thrombus formation and recanalization may be the main etiologic factor. Because of preoperatively undiagnosed thrombosis of the portal venous system, the operative mortality is high. The calcification of portal venous system with associated thrombosis is a significant finding and more attention should be devoted to detecting in patients undergoing liver transplantation.

## Author contributions

BP and SCL contributed to the planning and organization. BP and SCL collected clinical data and supervised the findings of this work. QH aided in the data collection and the supervision. BP and SCL analyzed the results and prepared the manuscript. All authors contributed to the article and approved the submitted version.

Conceptualization: Qiang He.

Data curation: Bing Pan.

Resources: Bing Pan, ShaoCheng Lyu.

Software: Bing Pan.

Supervision: Qiang He.

Validation: Qiang He.

Writing – original draft: Bing Pan.
